# Fish Consumption and Ischemic stroke in Southern Sweden

**DOI:** 10.1186/1475-2891-10-109

**Published:** 2011-10-11

**Authors:** Anna Oudin, Maria Wennberg

**Affiliations:** 1 Department of Public Health and Clinical Medicine, Umeå University, Umeå, Sweden

**Keywords:** Fish intake, fish consumption, stroke, ischemic stroke

## Abstract

**Background:**

The relationship between fish intake and stroke incidence has been inconsistent in previous Swedish studies. Here, we report the risk of stroke and fish intake in a cohort from southern Sweden.

**Findings:**

Data were obtained from an already available population based case-control study where the cases were defined as incident first-time ischemic stroke patients. Complete data on all relevant variables were obtained for 2722 controls and 2469 cases. The data were analyzed with logistic regression analysis. Stroke risk decreased with fat fish intake ([greater than or equal to] 1/week versus <1/month) in both men and women; adjusted pooled Odds Ratio (OR) 0.69, 95% Confidence Interval (CI): 0.54-0.89. However, stroke risk for women increased with intake of lean fish; adjusted OR 1.63 (95% CI: 1.17-2.28), whereas there was no association with men's lean fish intake; adjusted OR 0.97(95% CI: 0.73-1.27). Fish intake was self-reported retrospectively, yielding uncertain exposure assessment and potential recall bias. The findings regarding lean fish could be explained by recall bias if an individual's inclination to report lean fish consumption depended on both disease status and sex. The fact that the association between fat fish intake and stroke was similar in men and women does not support such a differential in recall.

**Conclusions:**

The results suggest fat fish intake to decrease ischemic stroke risk and lean fish intake to increase women's stroke risk. The inconsistent relationship between fish intake and stroke risk reported in previous studies is further stressed by the results of this study.

## Introduction

Ischemic stroke is one of the major causes of morbidity and mortality across the world. Many of the risk factors are associated to lifestyle, and in rich countries an increased burden of the disease are often observed in low socio-economic (SES) groups. It is therefore of great importance to identify lifestyle factors that might explain some of the variance in ischemic stroke risk between different SES groups.

The evidence for fish intake as a protective factor is less convincing for stroke than for cardiovascular disease in general [1-6]. The results seem to vary between populations with respect to geographical areas, sex and fish intake habits. A weakness in many epidemiological studies on fish consumption and stroke has been a failure to distinguish between different types of stroke and different types of fish. The effects of fish intake on human health has been debated; both beneficial (via omega-3 fatty acids) and adverse health effects (via the environmental pollutants methylmercury and PCB) are plausible, but there seem to be more scientific evidence for beneficial effects than for adverse effects [7]. In a review from 2003, American Heart Association stated that evidence was compelling enough to justify recommendations of at least two servings of fish, especially fat fish, per person and week [6]. The Swedish Food Administration recommends 2-3 meals of fish per week [8].

In a prospective case-control study within the Northern Sweden Health and Disease Study, we observed an increased risk of stroke in men reporting fish consumption more often than three times a week, as compared to less than once a month [9]. The association was similar for lean and fat fish consumption. No statistical significant association was found in women. Recently, lean fish intake, but not intake of other types of fish, was observed to reduce total stroke risk in Swedish women [10]. The disparities in results regarding sex-specific effects and type of fish (lean or fat) between the two Swedish studies are intriguing. We therefore aimed at studying the relationship between ischemic stroke and fish intake in an already available data material from southern Sweden.

## Materials and methods

Data stemmed from a data material collected to investigate the effects of air pollution on ischemic stroke risk, designed as a case-control study, where cases were identified as patients with incident first-time ischemic strokes between the years 2001 and 2006. The original study has been more extensively described elsewhere [11]. The study base was the population of the southernmost region in Sweden (Skåne), with a population of 1 243 329 on December 31, 2010 according to official statistics. Data on cases were obtained from the national stroke register (Riks-stroke), yielding high quality case assessment. All incident cases in Skåne who were born between 1923 and 1965 were included (N = 7244). Questionnaires were sent out in November 2008 to surviving cases (N = 4621) and to population controls, matched on birth year and sex (1:1).

The questionnaires contained questions on occupational history, lifestyle factors, residential environment, heredity and disease history. The response rate was about 70% among cases and 73% among controls. The variables used were: Birth year category (1923-1925,1926-1930,1931-1935,1936-1940,1941-1945,1946-1950,1951-1955,1956-1960,1961-1965), Marital status (Married, Divorced, Widowed or Neither of those), Education level (≤9 years, 10-12 years or >12 years), Birth country (Sweden, Nordic, Other), Municipality, Medication for hypertension (Yes/No), Atrial fibrillation (Yes/No), Smoking (Yes/No), Diabetes mellitus(Yes/No), Physical inactivity(Yes/No), Daily fruit intake (Yes/No), Body Mass Index (BMI), Lean fish intake (cod, butt, pike and perch were mentioned as examples of lean fish), and Fat fish intake (herring, salmon, trout, lavaret, salmon trout and eel were mentioned as examples of fat fish). Self-reported fish intake at the time the stroke had occurred was reported in the categories "Seldom/Never", "At least once a month" and "At least once a week", which we here renamed "<1/month", "1/month to <1/week" and "≥1/week". Data were analyzed with logistic regression analysis. The association between fish intake and ischemic stroke was modeled both separately for men and women and as pooled estimates. Effect modification by sex was investigated. For fish intake, tests for trend were done.

The final multivariate models contained the main variables Birth year category, Sex, Lean and Fat Fish Intake, and the following variables selected with backward selection technique: Medication for hypertension, Atrial Fibrillation, Education, Smoking, Diabetes mellitus and Fruit Intake. In order to increase comparability with the study by Wennberg and colleagues, [9] we produced models including the following variables: Diabetes mellitus, Hypertension, BMI and Smoking together with the main variables. Data were analyzed with SAS v 9.2. The figures were created with PASW Statistics 18.

## Results

**Figure 1:**
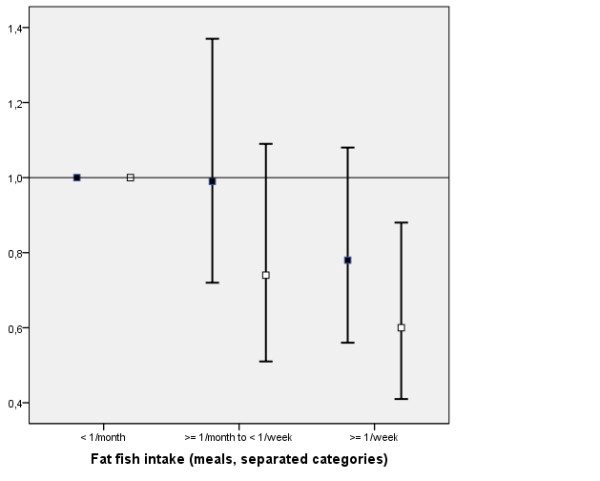
**Adjusted Odds ratios and 95% Confidence Intervals for Ischemic Stroke in association with Fat Fish intake**. Adjusted Odds ratios and 95% Confidence Intervals for Ischemic Stroke in association with Fat Fish intake for Men (black squares) and Women (white squares). The variables included into the adjusted models were: Birth year category, Sex, Lean Fish Intake, Fat Fish Intake, Medication for Hypertension, Atrial Fibrillation, Education, Smoking, Diabetes mellitus and Fruit Intake.

**Figure 2:**
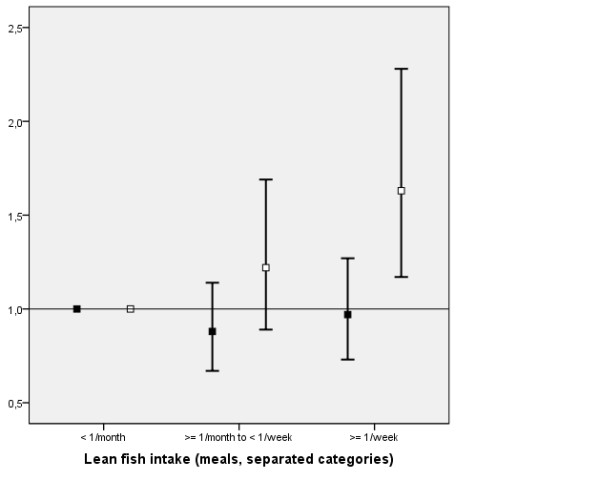
**Adjusted Odds ratios and 95% Confidence Intervals for Ischemic Stroke in association with Lean Fish intake**. Adjusted Odds ratios and 95% Confidence Intervals for Ischemic Stroke in association with Lean Fish intake for Men (black square) and Women (white square). The variables included into the adjusted models were: Birth year category, Sex, Fat Fish Intake, Lean Fish Intake, Medication for Hypertension, Atrial Fibrillation, Education, Smoking, Diabetes mellitus and Fruit Intake.

Descriptive data stratified for disease status, sex and exposure category is presented in Table 1. The fish intake effect seemed to differ between men and women regarding lean fish (p-value for effect modification = 0.06), but not fat fish (p > 0.5). Fat fish intake was associated with a decrease in stroke risk (≥ 1/week versus <1/month); adjusted Odds Ratio (OR) 0.69, 95% Confidence Interval (CI) 0.54-0.89 (pooled estimate for men and women), p for trend < 0.0001. In sex-specific analysis, fat fish consumption was associated with decreased risk of stroke in women, and there was a similar trend, although not statistically significant, in men (Figure 1). Lean fish intake, on the contrary, was in women associated with an increase in stroke risk with an OR of OR 1.63 (95% CI: 1.17-2.28), p for trend = 0.0005, whereas there was no association with men's lean fish intake; adjusted OR 0.97 (95% CI: 0.73-1.27) (Figure 2), p for trend = 0.60.

**Table 1:** Fish intake separated by disease status and sex.

		***Cases***			***Controls***		
			
		**Men N(%)**	**Women N(%)**	**Total N(%)**	**Men N(%)**	**Women N(%)**	**Total N(%)**
			
*Lean fish intake*	<1/month	190(13)	104(10)	294(12)	178(11)	127(11)	305(11)
	≥1/month to 1/week	660(46)	430(41)	1090(44)	738(47)	508(45)	1246(46)
	≥1/week	576(40)	509(49)	1085(44)	665(42)	506(44)	1171(43)
							
*Fat fish intake*	<1/month	123(9)	91(9)	214(9)	109(7)	67(6)	176(6)
	≥1/month to 1/week	604(42)	377(36)	981(40)	595(38)	393(34)	988(36)
	≥1/week	699(49)	575(55)	1274(52)	877(55)	681(60)	1558(57)

The crude effects were rather similar to the adjusted estimates, although the effect estimates for both lean and fat fish intake were weakened when not adjusted for each other (data not shown). In a model similar to the one used in the northern Sweden study (including the variables Diabetes mellitus, Hypertension, BMI, Smoking, Birth year category and Sex), the fish intake effect estimates were almost identical to those presented here (data not shown).

## Discussion

We did not replicate the findings from northern Sweden, where both lean and fat fish intake seemed to increase ischemic stroke risk in men but not in women [9]. On the contrary, lean fish intake was in this Southern Sweden cohort associated with an increase in ischemic stroke risk in women but not in men, whereas fat fish intake seemed to have a protective effect in both women and men.

A marker for fish consumption prior to stroke was obtained by asking cases to estimate their fish intake at the time before their stroke and by asking controls to estimate their fish intake the year their matched case had the stroke. The relevant time window for fish consumption to influence stroke risk is uncertain. Exposure measurement error depends on how well that marker for fish consumption reflects actual and relevant exposure, and will likely, given that reporting is non-differential, yield bias towards the null. Moreover, the amount, or weight of fish is not necessarily reflected by how often fish is consumed. Information on fish intake was self-reported retrospectively in the present study. Validations of the fish-intake variables were not feasible in this setting, but validations of some of the other retrospectively self-reported variables (Hypertension, Smoking, Diabetes mellitus and Atrial fibrillation) suggested that the accordance with register data was generally fair, which can likely be generalized to other self-reported variables in the study, such as the fish intake variables. Given that fish intake is known to be beneficial; it is possible that recall bias was present, but it is less likely that recall bias differ between the effect estimates of fat and lean fish intake. In order to create recall bias away from the null (and thus explain the findings regarding lean fish intake in women) an individual's inclination to report fish intake would have to depend both on disease status (case/control), sex and type of fish intake reported. Neither the findings regarding fat fish (similar in men and women), nor previous results from the southern Sweden data material, support such a differential in recall.

Data allowed only for one fish intake category among those who ate fish once a week or more often (≥1/week). Thus, the present study did not allow for distinguishing of effects in persons with high consumption of fish, in fact; fish intake once a week is less than what is recommended by the Swedish Food Administration. An association between ischemic stroke risk and fish intake was detected nevertheless, but it would have been desirable to better distinguish effects of high consumption of fish.

The observed effects regarding lean and fat fish consumption were weakened if not adjusted for each other. This would be expected under a causal inference assumption (assuming beneficial effects of fat fish and harmful effects of lean fish intake), since lean and fat fish intake are highly correlated.

A protective effect of fat fish, but not of lean fish, could theoretically be explained by the higher content of beneficial omega-3 fatty acids in fat fish and a higher content of environmental pollutants in lean than fat fish. For example, methylmercury (in Sweden found more often in lean than fat fish) is a potential explanation for the risk increase observed in frequent lean fish consumers. However, Wennberg et al. did not find an association between stroke risk and levels of methylmercury [9]. Other known pollutants in fish, like persistent organic pollutants, are found in higher levels in fat fish. Moreover, we are not aware of a plausible explanation of how lean fish could increase stroke risk in women, but not in men.

Despite the weaknesses regarding exposure assessment, the findings are intriguing and further stress the inconsistent relationship between fish intake and stroke risk observed in previous studies.

## Key results

Fat fish intake decreased the risk of ischemic stroke in both women and men. Lean fish intake increased ischemic stroke risk in women but not in men.

## List of abbreviations

OR: Odds ratio; CI: Confidence Interval; SES: Socio-economic

## Competing interests

The authors declare that they have no competing interests.

## Authors' contributions

AO collected the data, carried out the statistical analysis and did most of the writing. MW helped draft the manuscript. All authors read and approved the final manuscript.
